# Programmed Cell Death Ligand as a Biomarker for Response to Immunotherapy: Contribution of Mass Spectrometry-Based Analysis

**DOI:** 10.3390/cancers17061001

**Published:** 2025-03-17

**Authors:** Marco Agostini, Pietro Traldi, Mahmoud Hamdan

**Affiliations:** Istituto di Ricerca Pediatrica Città della Speranza, Corso Stati Uniti 4, 35100 Padova, Italy; m.agostini@unipd.it (M.A.); mhglaxo@gmail.com (M.H.)

**Keywords:** programmed cell death ligand, predictive biomarkers, immune checkpoint inhibitors, immunotherapy, mass spectrometry-based analysis, post-translational modifications

## Abstract

Immune checkpoint inhibition is a major component in today’s cancer immunotherapy. In recent years, the FDA has approved a number of immune checkpoint inhibitors (ICIs) for the treatment of melanoma, non-small-cell lung, breast and gastrointestinal cancers. These inhibitors, which target cytotoxic T-lymphocyte antigen-4, programmed cell death (PD-1), and programmed cell death ligand (PD-L1) checkpoints have assumed a leading role in immunotherapy. The same inhibitors exert significant antitumor effects by overcoming tumor cell immune evasion and reversing T-cell exhaustion. The initial impact of this therapy in cancer treatment was justly described as revolutionary, however, clinical as well as research data which followed demonstrated that these innovative drugs are costly, are associated with potentially severe adverse effects, and only benefit a small subset of patients. These limitations encouraged enhanced research and clinical efforts to identify predictive biomarkers to stratify patients who are most likely to benefit from this form of therapy. The discovery and characterization of this class of biomarkers is pivotal in guiding individualized treatment against various forms of cancer. Currently, there are three FDA-approved predictive biomarkers, however, none of which on its own can deliver a reliable and precise response to immune therapy. Present literature identifies the absence of precise predictive biomarkers and poor understanding of the mechanisms behind tumor resistance as the main obstacles facing ICIs immunotherapy. In the present text, we discuss the dual role of PD-L1 as a biomarker for response to immunotherapy and as an immune checkpoint. The contribution of mass spectrometry-based analysis, particularly the impact of protein post-translational modifications on the performance of this protein is underlined.

## Simple Summary

This review discusses the search for biomarkers in response to immune checkpoint inhibitors. The main body of the discussion focuses on one of these biomarkers, which has been approved by the Food and Drug Administration to stratify patients, who are candidates for this form of therapy. The text underlines the limitations of this biomarker, and current research efforts to discover and develop more efficacious biomarkers. The contribution of mass spectrometry to such efforts is emphasized. The review concludes by pointing out two main challenges to this form of therapy: First, developing treatments that could potentially expand the efficacy of immune checkpoint therapies to current non-responders, and second, understanding the mechanisms responsible for the heterogeneity of response and the development of resistance to this type of therapy.

## 1. Introduction

Immune checkpoint inhibitors targeting cell death ligand-L1(PD-L1), programmed cell death-1 (PD-1), cytotoxic T-lymphocyte antigen-4 (CTLA-4), and lymphocyte activation gene-3 (LAG-3) have substantially improved the cure and the management of several forms of cancer. The introduction of immune checkpoint inhibitors (ICIs) represented an important breakthrough in cancer therapy and demonstrated durable response in many tumor types, yet the majority of patients either failed to respond or developed resistance to these inhibitors. It has been demonstrated that this form of therapy benefits a small subset of patients, it has high costs, and can be accompanied by severe adverse effects. These limitations underline the urgent need for predictive biomarkers to identify patients who will benefit from such treatment. Expression level of the PD-1 receptor and PD-L1, as assessed by immunohistochemistry (IHC) is the first biomarker to be used to predict immunotherapy response [1,2]. Currently, there are three FDA-approved predictive biomarkers: Programmed cell death ligand-1 (PD-L1); microsatellite instability/defective mismatch repair (MSI/dMMR), and tumor mutational burden (TMB)), which are routinely used for patient selection for ICIs response in clinical practice. PD-L1 was the first FDA-approved predictive biomarker for non-small-cell lung cancer (NSCLC). Since then, the FDA has approved the same biomarker as a complementary diagnostic test for six additional tumor types. Currently, PD-L1 is the most investigated and clinically used predictive biomarker for ICIs therapy [3,4]. Microsatellite instability/defective DNA mismatch repair (MSI/dMMR) [5] was the second FDA-approved predictive biomarker for the pembrolizumab treatment of adult and pediatric patients with unresectable or metastatic solid tumors. TMB was approved in 2020 to monitor response to pembrolizumab (monoclonal antibody) in the treatment of adult and pediatric patients with unresectable or metastatic solid tumors [6].

**Figure 1:**
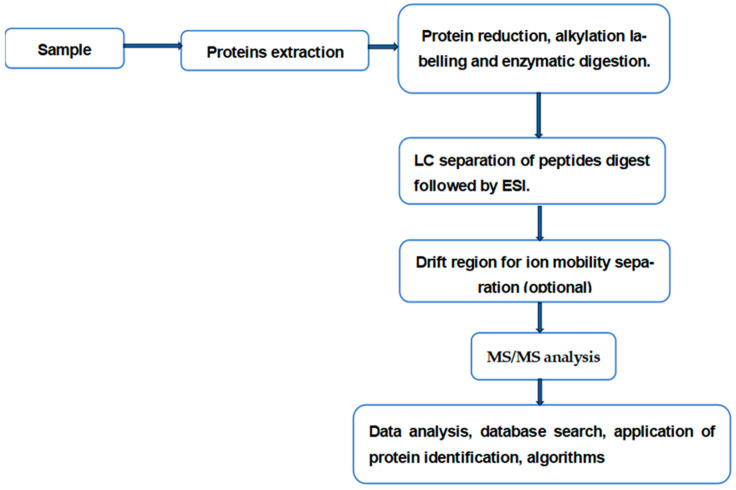
The main steps in a workflow commonly used for the analysis of protein mixtures. This experimental arrangement can be used in three different modes of analysis, depending on the sample to be analyzed: “bottom-up” method for digest of short peptides (5–20 amino acids); middle-down (20–50 amino acids); top-down (intact proteins). The main difference between the three methods is restricted to sample preparation and the choice of the LC components.

The use of predictive biomarkers for the stratification of melanoma patients is a representative example on how predictive biomarkers for immunotherapy can help clinicians choose the best therapeutic strategy. Melanoma has always been described as a highly lethal form of cancer [7,8]. In the last 12 years, several new therapeutic strategies have demonstrated an increase in survival in the metastatic and adjuvant settings [9,10]. One of these strategies is the use of ICIs, which resulted in long-term remissions for a subset of melanoma patients [11]. Despite the initial success of this form of therapy, to date, reliable biomarkers able to predict melanoma response to checkpoints inhibition are still lacking. It can be argued that the notable achievements in immunotherapies have not yet been matched by the development of precise predictive biomarkers able to identify the ideal target populations for ICIs therapy. It has to be emphasized that the role of these biomarkers in immune therapy is not limited to the classification of patients receiving ICIs into responders and non-responders. These predictive biomarkers perform additional functions, including monitoring treatment efficacy, disease progression, better understanding of the mechanisms behind acquired resistance to therapy, guide decision making regarding anti-tumor immunotherapy, and can contribute to a more informed design of clinical trials. Current literature describes various approaches to identify and validate potential predictive biomarkers. One of these approaches describes the use of gut microbiome to predict the response of lung and kidney cancer patients to immunotherapy [12]. Basically, this approach assesses the balance between two groups of gut bacteria (anti- or pro-inflammatory). The balance between these two functional groups can be used as a diagnostic indicator to evaluate the intestinal dysbiosis associated with survival in patients with lung and kidney cancers. A recent article on immunotherapy describes this biomarker together with others based on longitudinal blood-based biopsies and radiomics as dynamic biomarkers [13]. The same article underlines the difference in performance between these biomarkers and their tissue-based counterparts (such as tumor gene signatures, tumor antigen presentation, or tumor microenvironment profiles). It is useful to bear in mind that there are hundreds of proteins involved in the immune response to cancer [14,15]. Quantifying those immunomodulatory proteins and their eventual characterization, particularly associated post-translational modifications (PTMs), should present an opportunity for a better understanding of the response to treatments and likely predictive biomarkers to such response. Recent advances in mass spectrometry (MS), liquid chromatography (LC), and sample preparation, including protein labeling, renders MS-based proteomics a key player in the analysis of immune checkpoints. These analyses can provide relevant information on PTMs, protein quantification, subcellular protein localization, and cell signaling,

Liquid chromatography (LC) coupled to tandem mass spectrometry (MS/MS) is the principal platform for the analysis of proteins present in complex biological samples (Figure 1). Enzymatic peptides within a protein digest are separated by LC and introduced into an electrospray ion source, where multiply charged gas-phase peptide ions are formed. These ions are scanned by the first mass analyzer to be transmitted into a collision region. Product fragment ions are scanned by a second mass analyzer and transmitted to ion detector and data analysis. The separation capability of this system can be enhanced by adding another dimension to the separation process. This can be achieved by including an ion mobility component in the path of the ions prior to their fragmentation. This ion mobility component enhances the structural information acquired by LC-MS/MS, in particular isobaric structures. This is due to the fact that ion mobility separates gas-phase ions according to their mass/charge ratio, size, and shape [16]. In DDA, the ions mixture is scanned by the first *m*/*z* analyzer (MS1), which identifies the most abundant *m*/*z* values to be injected into a collision cell for fragmentation. Scanning MS2 transmits the resulting fragment ions to detection and data elaboration. DIA is considered a major development in the field of MS-based proteomics. This method has the capability to systematically sample all peptides in a given *m*/*z* range, allowing an unbiased acquisition of MS/MS data. This capability greatly reduces missing values and significantly enhances quantitative accuracy, precision, and reproducibility compared to many traditional methods [17]. Sequential window acquisition of all theoretical mass spectra (SWATH-MS) is a variant of data-independent acquisition [17,18]. In DIA acquisition mode, MS1 is used to transmit a relatively wide (*m*/*z* 25), slightly overlapping precursor isolation windows. Basically, the mass range in this analysis is usually between 400 and 1200 *m*/*z* can be examined by transmitting 32 precursor ion windows. Such a fragmentation scheme, involving wide precursor ion windows will inevitably lead to the co-fragmentation of many co-eluting peptides concurrently selected in these windows and ultimately to highly multiplexed and complex MS/MS spectra. The main innovative feature of DIA is in its data analysis strategy capable of handling the complexity of the generated spectra. Sequential window acquisition of all theoretical mass spectra (SWATH-MS) is the most diffused version of DIA. It has been developed to enhance MS/MS capability for protein identification in complex samples. Transmitting relatively wide *m*/*z* windows rather than a single *m*/*z* by MS1 allows unbiased acquisition of MS/MS data, and reduces the number of ions, which can be missed (missed values) in traditional methods [17]. A number of works reported that the use of this mode of acquisition, enhances quantitative accuracy, precision, and reproducibility. It should be pointed out that the strength of SWATH-MS is based on a more tolerant *m*/*z* resolution of the first analyzer combined with fairly sophisticated and complex software. On the hardware side, the method does not add any novelty, apart from the need to use high-speed, high-resolution instruments together with long LC gradients [17,18].

## 2. Discussion

### 2.1. PD-L1 Expression as Predictive Biomarker to ICIs Therapy

Existing cancer biomarkers perform different roles in the world of oncology. These roles can be diagnostic, prognostic, or predictive. Data accumulated over the last three decades regarding cancer biomarkers demonstrate a vast variety of these biomarkers, including proteins, DNA, RNA, metabolites, and gene and protein signatures. It is generally agreed that the ideal biomarker should have a number of characteristics, including high sensitivity, high specificity, simple and reproducible detection method, and easy to validate clinically, including its inclusion in large-scale clinical trials. It can be said that the therapeutic leap in immune therapy, which started with the approval of ICIs inhibitors for the treatment of various forms of cancer, has not been matched by the discovery and validation of precise predictive biomarkers for reliable prediction of the response to such therapy. Table 1 gives both biomarkers approved by the FDA and potential biomarkers in various phases of testing and validation. In the present text, an attempt is made to discuss the role of PD-L1 as a leading biomarker in response to ICIs therapy.

### 2.2. The Role of PD-L1 as Immune Checkpoint

**Figure 2:**
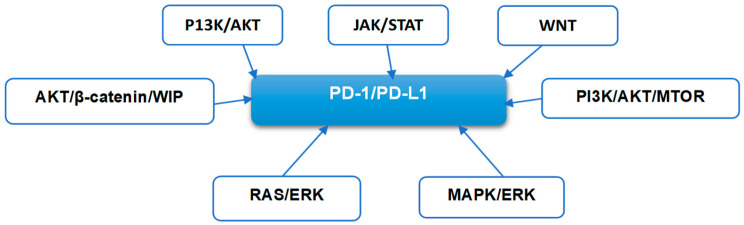
Signaling pathways, which are known to interact with PD-1/PD-L1 axis. The figure is partially based on data in [31].

Programmed death ligand 1 (PD-L1, also known as B7-H1 or CD274) in combination with programmed cell death-1 (PD-1) plays a relevant role in tumor escape of immune surveillance. This protein performs its functions through the PD-1/PD-L1 axis. Numerous studies [1,28,29] have demonstrated that interaction between PD-1 and its ligand, PD-L1, negatively regulates immune response and reduces the surveillance capability of the immune system toward various forms of cancer. This regulatory role by PD-1/PD-L1 results in the inhibition of the activity of effector T-cells while enhancing the function of immunosuppressive regulatory T-cells (Tregs). Blocking this interaction through the use of monoclonal antibodies is gaining an important role in immune therapy. The expression of PD-L1 in different cancers can be regulated by a number of transcriptional factors, including HIF-1, STAT3, NF-κΒ, and AP-1 and certain oncogenic pathways such as JAK/STAT, RAS/ERK, or PI3K/AKT/MTOR (see Figure 2). The activation of PI3K/AKT/mTOR pathway is known to be closely related to PD-L1 expression and can iimpact the tumor immune microenvironment [30,31]. Over 20 years ago [32], it was demonstrated that the PI3K/Akt/mTOR signaling pathway can influence the expression of PD-L1. Clinical implications of the interaction between the PD-1/PD-L1 and PI3K/AKT/mTOR pathway have been reviewed, focusing on patients suffering from non-small-cell lung cancer (NSCLC) [31]. One of the relevant questions raised by this review is the viability of a therapeutic strategy combining PI3K/AKT/mTOR pathway inhibitors with PD-1/PD-L1 inhibition. In this well-designed review, and taking NSCLC as an example, the authors discussed in some detail the various components of the PI3K/AKT/mTOR signaling pathway and described their functions in driving carcinogenesis and the suppression of antitumor activity. The same review discussed the interaction between this pathway and PD-L1, and how such interaction influences the expression of this checkpoint. In a much earlier work [32], it was shown that the activation of common oncogenic pathways such as JAK/STAT, RAS/ERK, or PI3K/AKT/MTOR, as well as treatment with cytotoxic agents can affect tumoral PD-L1 expression.

### 2.3. PD-L1 as Predictive Biomarker in Response to ICIs Therapy

The expression level of PD-L1 in tumor tissues of various cancer patients is considered the main predictive biomarker for the identification of patients who are likely to receive therapeutic benefit from anti-PD-1/PD-L1 therapy. This statement is based on various studies, which reported that in some forms of cancer, the high expression level of PD-L1 indicates a better therapeutic effect for patients receiving anti-PD-1/PD-L1 therapy [33,34,35]. PD-L1 level of expression as a predictive biomarker in response to immunotherapy has been in use for a number of years, yet there is still a strong debate regarding its predictive efficacy. Such debate has been sustained by a number of studies showing that the expression of PD-L1 in some NSCLC patients was low or even negative, but the response to anti-PD-1/PD-L1 treatment was better [36,37] on the other hand, some patients had higher expression of PD-L1, and the therapeutic effect of anti-PD-1/PD-L1 therapy was poor.

Assessment of PD-L1 level of expression in patients is normally performed using immunohistochemical (IHC) staining. In the last few years, the FDA and European Medicines Agency (EMA) have approved four PD-L1 IHC antibodies along with their associated staining protocols conducted on two manufacturer-specified platforms. Despite its prominent predictive role in a number of cancer forms, PD-L1 is a single marker, liable to a number of confounding factors that challenge its reliability and its predictive value. For example, PD-L1 is known to experience extensive glycosylation, a post-translational modification in the extracellular domain. Such modification is known to impact on the stability of this protein and its interaction with the immune system. To reduce the interference by this PTM, a protocol incorporating pre-treatment of glycosylation removal before IHC staining has been developed [38,39]. The use of such protocol improved notably the detection of PD-L1 across a number of tumors, including colon, lung, breast, pancreas, and prostate. Furthermore, the application of this protocol resulted in a stronger correlation between PD-L1 detection and patient responses to ICIs treatment [40,41,42,43]. Another confounding factor affecting the reliability and predictive capability of PD-L1 staining can be provoked by the method of sampling. In other words, if the method of sampling does not accurately reflect the heterogeneity of PD-L1 expression within a given tumor or between different tumors, then the accuracy and the predictive value of PD-L1 is likely to be less representative of the response to therapy. It should be emphasized that the influence of PD-L1 heterogeneity is mainly observed in tissue analysis. To reduce the effect of this limitation, a number of recent studies used liquid biopsy samples for the assessment of PD-L1 expression on circulating tumor cells (CTCs).

### 2.4. Assessment of PD-L1 Expression on Circulating Tumor Cells

Over the last decade, various studies have demonstrated that a wide range of molecular and cellular entities can be shed into various body fluids. These circulating analytes are mainly derived from diseased sites, and may include circulating tumor cells (CTCs), cell-free DNA (cfDNA), circulating tumor DNA (ctDNA) proteins, peptides, and various metabolites. Analysis of these body fluids (liquid biopsies) can furnish crucial information on these circulating entities and associated diseases. The increasing use of liquid biopsy sampling instead of tissue sampling may reduce the effect of heterogeneity of PD-L1 expression within a given tumor or between different tumors, thus enhancing the detection reliability of PD-L1 as a predictive biomarker in response to PD-1/PD-L1 therapy. The substantial increase in the use of liquid biopsy sampling in cancer profiling can be attributed to a number of advantages, which this method offers, including noninvasive serial sampling, which allows almost real-time monitoring of the disease, and the capability of this method to capture the heterogeneity of the disease, a capability which is missing in the tissue sampling method. A number of studies reported the assessment of PD-L1 expression on circulating tumor cells within various liquid biopsies; Table 2 lists a number of these studies [44,45]. The investigations listed in Table 2 use liquid biopsies rather than tissues to determine PD-L1 expression on circulating CTCs. The main limitation of this approach is the low abundance of CTCs in blood samples. A number of studies have shown that a patient sample may not contain more than 10 of these circulating cells in a background of 10^6^ white blood cells and 10^9^ red blood cells [46,47,48]. Moreover, CTCs are known to form cellular aggregates with cells like fibroblasts and platelets. These aggregates have been reported to spread to more distant sites in the body relative to their isolated CTC counterparts [49,50].

### 2.5. MS-Based Analysis of PD-L1 Post-Translational Modifications

**Figure 3:**
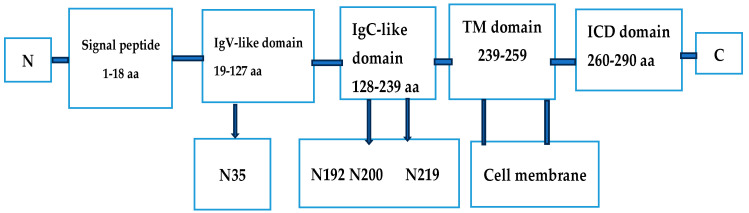
PD-L1 domains and glycosylation sites. The figure shows 4 glycosylation sites at the indicated asparagine residues (N), N35, N192, N200, N219. Figure based on data reported in [40].

Both PD-1 and PD-L1 can experience various forms of post-translational modifications (PTMs), some of which are listed in Table 3. For a number of years, genetic, transcriptional and post-transcriptional regulation were considered the main regulators of the PD-L1/PD-1 pathway. More recently, the role of post-translational modifications started to attract more attention. N-linked glycosylation is one of these modifications. Glycosylation is one of the most common PTMs, in which polysaccharides are transferred to specific amino acid residues in proteins by glycosyltransferases. There is strong evidence that glycosylation is essential for the unfolding of various functional activities in organisms, such as playing a role in the regulation of protein function, cell adhesion, and immune escape. It is well known that both PD-L1 on tumor cells and PD-1 on tumor-specific T-cells undergo extensive N-linked glycosylation, which is essential for the stability and interaction of both proteins [55,56]. Some sites of N-linked PTMs are given in Figure 3.

The impact of glycosylation and other PTMs on cancer therapy has been investigated by a number of studies [67,68,69]. High-resolution mass spectrometry was used to investigate glycopeptides following their enrichment by anion exchange-mediated methods [70]. The authors used nano-LC coupled to high-resolution MS to detect glycopeptides containing polyLacNAc motifs in melanoma cells; the two proteins, integrin and tetraspanins, were found substantially modified with these structures. The detection of these structures can be complicated due to their low abundance, high structural heterogeneity, and chemical–physical properties. The same structures are known to contribute to cancer resistance against T-cell killing [70,71]. PolyLacNAc motifs also contribute to an increased stability of PD-L1, leading to an enhanced interaction with PD-1, resulting in reduced cytotoxic T-cell responses in cancer [72]. Sample preparation for MS analysis and enrichment methods have been fully described in the original article [70], and we see no benefit in repeating here. However, we retain the following observations as relevant. A central theme in the investigation is the enrichment efficiency of glycopeptides containing polyLacNAc motifs. Five anion exchange-based matrices for enriching intact glycopeptides were evaluated before choosing the best assay for such analyses. Second, the MS measurements were performed on a high-resolution instrument, using nano electrospray ionization and data-dependent acquisition mode (DDA). In this mode of acquisition, high signal ions (precursor ions) within a relatively narrow scan window are selected for fragmentation in the MS/MS phase. In their analysis, the authors reported the selection of twenty peptides for collision-induced dissociation. In another recent study, mass spectrometry was used to investigate N-linked and O-linked glycosylation of PD-1. It is interesting to note that the PD-1 structure has been investigated for over twenty years, yet this is the first study to identify O-linked glycosylation sites located in the stalk region of the protein [61]. We should point out that the identification of PTM sites is normally performed using LC-MS/MS; in this study, the authors simply used LC/MS to identify the O-linked glycosylation sites. The success of these measurements was facilitated by the use of O-protease digestion and intact mass analysis. This method is simpler and less costly than analysis in which MS/MS has a central role. That said, the application of this simple approach is limited to the mucin-type O-linked glycosylation that the O-protease derived from Akkermansia muciniphila is able to digest [73].

Ubiquitination is another prevalent post-translational modification, which affects various classes of proteins including checkpoints. This post-translational modification is considered an important factor in the regulation of PD-L1. Various E3 ubiquitin ligases have been identified to ubiquitinate PD-L1, leading to its proteasomal degradation, which impacts negatively on the immune escape capabilities of tumor cells. Under the action of these enzymes, ubiquitin can be linked to different lysine residues, and such linkage can be single or multiple (polyubiquitin chains). This modification is known to play a regulatory role in various biological processes in eukaryotic cells, including cell cycle, gene transcription, signal transduction, and in some mechanisms of resistance in cancer immunotherapy [74,75,76]. A detailed discussion of the various mechanisms of resistance and the role of ubiquitination in such mechanisms has been described by these works. One of these mechanisms underlines the role of ubiquitination in regulating the WNT/β-catenin signaling pathway. This pathway comprises a family of proteins that regulates key aspects of embryonic development, cell differentiation, proliferation, and adult stem cell homeostasis. In combination with various events, including inhibition of T-cell activation, there was increased indoleamine 2,3-dioxygenase 1 (IDO1) activity and upregulation in the PD-L1 level. This signaling pathway can regulate resistance to immune checkpoint inhibitors, which suggests that the inhibition of the WNT/β-catenin pathway may render immunotherapy against certain forms of cancer more effective. The use of MS-based investigations can give relevant information on the role of ubiquitarian in various diseases, including some forms of cancer. However, this analytical approach can be challenged by certain characteristics of this PTM. One of the challenges is to ensure that an LC-MS/MS method can provide useful information on branched ubiquitin chains. Such challenge can be supported by the following considerations: polyubiquitin chains are often assembled by accumulative attachment of ubiquitin to any of the seven lysine residues (K6, K11, K27, K29, K33, K48, and K63). This accumulative ubiquitination enhances the structural diversity through the formation of various topologies of different lengths and different branches. Global analysis of these chains in yeast over twenty years ago has revealed intense complexity, where all seven lysine residues can be used for the formation of ubiquitin–ubiquitin linkages [76]. The length and the structural diversity of these chains render the use of some well-established LC-MS/MS methods impractical (e.g., the popular bottom-up method). That said, ubiquitin chain enrichment in combination with the middle-down LC-MS/MS method was used to characterize branched ubiquitin chains present in human cell extracts [77,78].

Methylation is another PTM which is known to impact on the functions and interactions of PD-1/PD-L1. A recent study investigated PD-L1 methylation and its association with resistance to anti-PD-L1 therapy [79]. The authors reported that lysine in position 162 of PD-L1 was methylated by histone lysine methyltransferase 7 (SETD7). The same study suggested that methylation of amino acid 162 was a negative predictive marker for anti-PD-1 treatment in patients with non-small-cell lung cancer, and methylated PD-L1/PD-L1 ratio was an accurate biomarker for predicting response to anti-PD-(L)1 therapy. The authors used a host of analytical methods, including immunohistochemistry, immune blotting, immunofluorescence, deglycosylation, enzyme-linked immunosorbent assay (ELISA), real-time PCR, SDS–polyacrylamide gel electrophoresis, and LC-MS-MS. Prior to performing LC-MS/MS analysis, proteins were separated by SDS–polyacrylamide gel electrophoresis and protein bands were excised and subjected to standard preparations outside the mass spectrometer (reduction, alkylation, and digestion). The MS/MS data were acquired on a high-resolution mass spectrometer, operated in data-dependent acquisition mode.

In another study, the correlation between PD-L1 methylation and its level of expression in melanoma was assessed [80]. The authors compared the methylation level in hundreds of melanoma samples with that in 14 normal skin samples. This investigation reported that hypermethylation of PD-L1 in melanoma is associated with decreased PD-L1 expression and shorter patient overall survival. Furthermore, the methylation of this PD-1 ligand can be considered a significant independent prognostic factor of overall survival. The authors also reported the identification of key CpG loci regulating PD-L1 expression, and that such level of expression can be altered by using DNA hypomethylating agents such as 5-azacytidine. The authors reported that after 72 h, the treated melanoma cells showed a significant increase in PD-L1 mRNA expression compared to control samples treated with the same agent. These results indicate that PD-L1 methylation is involved in the regulation of PD-L1 expression. Considering the limited number of the investigated samples, in particular the number of controls, such regulation cannot be interpreted as an independent predictor of overall survival in melanoma, as suggested by the authors. A recent study examined the biological role of PD-L1/PD-L2 methylation and its association with clinicopathological features in pancreatic ductal adenocarcinoma (PDAC) [81]. The authors analyzed data on PD-L1/PD-L2 methylation and mRNA expression in PDAC cohorts obtained from the Cancer Genome Atlas and International Cancer Genome Consortium. The correlation between PD-L1 promoter methylation and PD-L1 expression was further validated in an independent validation cohort using DNA sequencing and immunohistochemistry. The main conclusion of this study was that hypomethylation of the PD-L1 promoter was strongly associated with upregulated PD-L1 expression and shorter overall survival in PDAC.

PD-L1 methylation and its impact on the interaction of PD-L1/PD-1 has been explored in another investigation [79]. These authors reported that methylation of PD-L1 at lysine 161 could control PD-1/PD-L1 interaction with the immune system, resulting in enhanced suppression of T-cell activity controlling cancer immune surveillance. The two studies cited here investigated the role of PD-L1 methylation in two different tumors, PDAC [81] and NSCLC [79]. The main conclusion of the latter study is that PD-L1 K162 methylation tumors were resistant to anti-PD-L1 and anti-PD-1 therapy. Furthermore, the same methylation served as a critical step for PD-1/PD-L1 interaction and as a negative predictive biomarker for anti-PD-1 treatment in patients suffering from NSCLC. Checkpoints which are the main target of ICIs therapy experience various PTMs, some of which are extensive (e.g., glycosylation in the case of PD-L1 and PD-1). It can be said that there is unanimous agreement that these modifications impact directly on the function of these checkpoints and on their reaction to the therapy. The main method of analysis of these modifications is MS-based proteomics. However, these highly informative data generated by this method are rarely combined with those generated by transcriptional, genomic, and epigenomic datasets. Such a combination is crucial for a deeper understanding of the biology of these modified checkpoints, their interaction with the immune system, and the impact of their modified structures on certain signaling pathways. The absence of such data integration may be partially responsible for the limitations associated with the approved predictive biomarkers.

## 3. Some Predictive Biomarkers Under Investigation

Table 1 lists a number of potential predictive biomarkers in response to immune checkpoint inhibitors (ICIs). These markers are still in various phases of evaluation, and there are still few clinical data on the efficacy of these potential biomarkers. That said, there are various works on the mechanisms of action and their role in resistance to ICIs therapy, and their association with certain adverse events of the same therapy. Two of these biomarkers are briefly considered below.

### 3.1. Protein Tyrosine Phosphatase Receptor T (PTPRT) as a Potential Biomarker

The human genome encodes 107 protein tyrosine phosphatases (PTPs); based on the amino acid sequence similarity of their catalytic domains, these PTPs have been divided into four classes [82]. PTPRT belongs to receptor protein tyrosine phosphatases, which contains 21 members [83]. Cancer genomic studies provided strong evidence that many members of this group of proteins function as tumor suppressor genes; such a claim is based on results showing that the majority of PTP mutations that have been identified in human cancers are loss-of-function mutations [83]. Discussing in more detail the role of these proteins in some human cancers is outside the scope of this review. Instead, we simply want to draw attention to some works that explored the role of PTPRT as a potential biomarker in response to ICIs therapy. In a recent investigation, the authors used mass spectrometry, Western blot, immunofluorescent staining, and next-generation sequencing (NGS) to investigate the response of PTPRT-deficient non-small-cell lung cancer to anti-PD-1therapy [81]. This study concluded that NSCLC patients who are PTPRT-deficient are likely to benefit from anti-PD-1 therapy. The same study reported that the loss of the same protein is a highly promising predictive marker independent of PD-L1 expression for anti-PD-1/PD-L1 therapy.

### 3.2. Gut Microbiome Changes

A growing body of evidence suggests that the baseline composition of a patient’s gut microbiota and its dysbiosis are correlated with the outcome of ICIs immunotherapy. Multiple clinical studies have demonstrated that the gut microbiome is an important regulator of systemic immune reactions and is involved in the response to ICI immunotherapy [84,85,86]. Existing literature leaves no doubt that ICIs therapy is playing a major role in the treatment of various forms of cancer. This form of treatment, however, is implicated in adverse effects. Collectively termed as immune-related adverse events (irAEs), these events can affect various organs, such as the heart, lungs, liver, and the gastrointestinal tract [87,88]. Gut microbiota refers to complex and diverse microorganisms residing in our intestines. It is now known that changes or shifts in the baseline composition of gut microbiota that present during the healthy state in an individual may signal an early warning of predisposition to multiple pathological conditions. It has been reported that shifts in the baseline composition can be influenced by a number of factors, including age, diet, geographic location, smoking, exercise, infections, and antibiotic administration [89]. The multiplicity of factors that impact the composition of gut microbiota renders its use as a predictive biomarker in response to ICIs therapy rather problematic. Despite this limitation, to date, most of the studies in this area have focused on the baseline microbiota composition as a predictive marker of response to ICIs. However, few studies attempted the use of bacterial metabolites rather than bacterial composition. This approach is based on the hypothesis that despite the variation in microbiota composition among individuals and over time, the gut microbiome metabolic pathways are what remain stable and mediate their interaction with the immune system to modify the response to ICIs. To date, most studies on the correlation between gut microbiome and immunotherapy focused on the potential of changes in this microbial community as predictive biomarkers for the response to ICIs therapy. However, few of these studies have highlighted the role of such changes in various adverse events associated with the same therapy. The complex heterogeneity of the human microbiome calls for large-scale analysis, employing different high-throughput omics techniques. Metagenomics, metabolomics, metatranscriptomics, and metaproteomics are in the forefront of such meta-analysis. Metaproteomics was first used over 20 years ago for a large-scale characterization of proteins present in environmental microbiota at a given point in time [90]. In their study, the authors used two-dimensional gel electrophoresis (2DE) for protein separation, followed by mass spectrometry identification of the individual proteins. Although this work is justly considered pioneering, it has two defects: low resolution and low throughput. This platform of analysis could detect about 2000 proteins, while current LC-MS/MS methods are capable of detecting a far higher number of proteins in a similar medium. The workflow to perform metaproteomic analyses is depicted in Figure 1. However, the large-scale analysis and the complexity of the gut microbiome necessitate optimization of both the LC and the MS. On the LC side, 1D-LC with long analytical columns and stationary phases with small particle sizes, which allow separating the complex metaproteomic peptide mixtures with high resolution using long LC gradients [91]. For this type of analysis, high resolution, high mass accuracy mass spectrometers are necessary.

Metagenomics and metaproteomics are the main techniques in the analysis of the human gut microbiome. Data generated by both techniques give crucial information on the proteomic and genomic composition of the microbiome at the time of analysis., Accurate information on the function of the detected proteins are likely to be provided by metaproteomics rather than by metagenomics. Such likely difference in performance can be partially supported by the following consideration: Functions performed by proteins within the investigated community are directly measured by proteomic analysis. On the other hand, not all DNA (genes) are transcribed into RNA, and not all RNA transcripts are subsequently translated into proteins. PTMs are known to play an important role in modulating protein activity within the microbiome. Metaproteomics has the capability of furnishing much needed information on these modifications [92,93].

## 4. Future Perspectives and Conclusions

The introduction of immune checkpoint inhibitors (ICIs) over a decade ago represented a therapeutic leap in the treatment of various forms of cancer. Clinical and research data generated within the same period, together with numerous clinical trials, revealed a number of limitations of this highly promising therapy. The high costs and associated serious adverse events of these inhibitors emphasized an urgent need for a reliable stratification of candidate patients. The last few years have witnessed intense research activities to discover and validate predictive biomarkers in response to ICIs therapy. In the present text, we focused on PD-L1 as a representative example of approved biomarkers. Based on the works reviewed in the present text, the following observations can be made: Most existing literature on ICIs confirm that this therapy benefits a small subset of cancer patients. This means that for ICIs to benefit a larger number of patients, it is not sufficient to discover more effective predictive biomarkers. The discovery of such biomarkers has to proceed in parallel with the discovery of a new generation of ICIs. This objective has two main challenges on its way. First, developing treatments that could potentially expand the efficacy of immune checkpoint therapies to current non-responders remain a significant challenge. Second, understanding the mechanisms responsible for the heterogeneity of response and the development of resistance to this type of therapy remain one of the main challenges for future research. Winning both challenges require more coordinated research efforts to generate more accurate proteomic, genomic, epigenetic, and transcriptional data, as well as more accurate identification of the various signaling pathways and certain PTMs, which regulate the function of the targeted checkpoints. These efforts have to be supported with large and well-designed clinical trials to test and validate both biomarkers and new therapeutic drugs.

## Figures and Tables

**Table 1:** Predictive biomarkers approved by the FDA for the assessment of response to ICIs therapy and other potential biomarkers in various phases of testing and validation.

FDA Approved Predictive Biomarkers	Some Potential Predictive Biomarkers Under Investigation.	Ref.
1. Programmed Death Ligand 1 (PD-L1) expression on tumor cells 2. Microsatellite Instability/Defective Mismatch Repair (MSI/dMMR) 3. Tumor Mutational Burden (TMB)	Gut microbiome.	[12]
	DNA damage response (DDR) gene alterations.	[19]
	Targeting DNA damage response pathways in cancer	[20]
	MHC-I genotypes.	[21]
	beta-2-microglobulin (B2M) deficiency.	[22]
	*POLE* mutations and *JAK1/2* mutations.	[23,24]
	Plasma biomarkers.	[25]
	POLE/POLD1 mutations.	[26]
	Loss of protein tyrosine phosphatase receptor type (PTPRT).	[27]

**Table 2:** Studies describing assessment of PD-L1 expression on circulating tumor cells (CTCs) and its role as predictive biomarker in response to PD-1/PD-L1 therapy.

Ref.	Investigated Disease	Method of Detection/ Sample	Conclusions/Comments
[51]	Advanced melanoma	Multiparametric flow cytometry Blood sample	A pilot study in which blood samples were collected from patients with metastatic melanoma receiving pembrolizumab (monoclonal antibody). Detectable PD-L1+CTCs were found in 64% of the patients. These patients had significantly longer progression-free survival (PFS) compared with patients with PD-L1− CTCs.
[52]	Hepatocellular carcinoma Esophageal cancer Gastric cancer Neuroendocrine carcinoma Colorectal cancer Pancreatic cancer	Immunofluorescence Blood sample	One of the conclusions of this study is that high PD-L1 expression on CTCs could influence response in patients receiving PD-1/PD-L1 therapy. Patients with high PD-L1 expression prior to treatment had a higher response rate to the same therapy as well as longer progression-free survival (PFS) and overall survival (OS).
[53]	Extensive-stage small-cell lung carcinoma (ES-SCLC). Limited-stage small cell lung carcinoma (LS-SCLC).	Immunofluorescence staining Blood and tissue samples.	Here, 43 patients were enrolled, 6 of them with ES-SCLC, 37 with LS-SCLC disease, and 10 healthy donors. This study concluded that correlation between samples derived from a limited number of ES-SCLC and LS-SCLC patients revealed a strong correlation between PD-1 expression on T-cells and PD-L1-expressing circulating CTCs. The same study reported that patients with high percentages of both CD3+CD8+PD-1+ T-cells and PD-L1+ CTCs had a survival advantage when treated with ICIs therapy.
[54]	Advanced non-small-cell lung cancer (NSCLC)	Immunofluorescence/ immunohistochemical staining Blood and tissue samples	This study examined PD-L1 expression in tissue and on circulating CTCs from a limited number of NSCLC patients. The authors reported that circulating CTCs released a higher detection rate of PD-L1 expression than tumor tissues. Patients with PD-L1 expression on tissue or CTCs had a median progression-free survival (mPFS) significantly longer than those without PD-L1 detection.

**Table 3:** Post-translational modifications of various amino acids of PD-1 and PD-L1.

Protein	Type of PTM	Sites of Modification	Reference
PD-1	N-linked glycosylation	N49, N58, N74, N116	[57]
	Phosphorylation	S261 Y223, T248	[58]
	ubiquitination	K233	[58,59,60]
	O-linked glycosylation	T153, T168, S157, S159	[61]
PD-L1	N-linked glycosylation	N35, N192, N200, N219	[62]
	Methylation	K75, K89, K105, R113, K162, R212	[63]
	phosphorylation	S176, T180, S184, S195, T210	[64,65]
	Acetylation	K263	[66]

## Abbreviations

ICIs	immune checkpoint inhibitors.
PD-L1	cell death ligand-L1
PD-1	programmed cell death-1
CTLA-4	cytotoxic T-lymphocyte antigen-4
LAG-3	lymphocyte activation gene-3
NSCLC	non-small-cell lung cancer
TMB	tumor mutational burden
FDA	Food and Drug Administration
dMMR	defective DNA mismatch repair
MSI	microsatellite instability understudied
PTMs	post-translational modifications
LC-MS/MS	liquid chromatography-tandem mass spectrometry
IHC	immunohistochemistry
B2M	beta-2-microglobulin
ESI	electrospray ionization
EMA	European Medicines Agency
CTCs	circulating tumor cells
ctDNA	circulating tumor DNA
PFS	progression-free survival
mPFS	median progression-free survival
DCR	disease control rate
ES-SCLC	extensive-stage non-small-cell lung cancer
DDA	data dependent acquisition mode
IDO1	indoleamine 2,3-dioxygenase 1
ELISA	enzyme-linked immunosorbent assay
real-time PCR	real-time polymerase chain reaction
qPCR	quantitative polymerase chain reaction
PTPRT	protein tyrosine phosphatase receptor T
cGAS	cyclic guanosine monophosphate–adenosine monophosphate synthase
STAT3	signal transducer and activator of transcription 3
2DE	two-dimensional gel electrophoresis (2DE)
